# Exposure to the environmental pollutant bisphenol A diglycidyl ether (BADGE) causes cell over-proliferation in *Drosophila*

**DOI:** 10.1007/s11356-020-08899-7

**Published:** 2020-04-28

**Authors:** Michael J. Williams, Hao Cao, Therese Lindkvist, Tobias J. Mothes, Helgi B. Schiöth

**Affiliations:** 1 Department of Neuroscience, Functional Pharmacology, Uppsala University, Husargatan 3, Box 593, 75 124 Uppsala, Sweden; 2 Institute for Translational Medicine and Biotechnology, Sechenov First Moscow State Medical University, Moscow, Russia

**Keywords:** Whole transcriptome sequencing, Environmental pollutant, Cell cycle, Hematopoiesis, Hemocyte

## Abstract

Bisphenol A diglycidyl ether (BADGE), a derivative of bisphenol A (BPA), is widely used in the manufacture of epoxy resins as well as a coating on food containers. Recent studies have demonstrated the adverse effects of BADGE on reproduction and development in rodents and amphibians, but how BADGE affects biological activity is not understood. To gain a better understanding of the biological effects of BADGE exposure during development, we used the model organism *Drosophila melanogaster* and performed whole transcriptome sequencing. Interestingly, when *Drosophila* are raised on food containing BADGE, genes having significantly increased transcript numbers are enriched for those involved in regulating cell proliferation, including DNA replication and cell cycle control. Furthermore, raising larvae on BADGE-containing food induces hemocyte (blood cell) over-proliferation. This effect can be stimulated with even lower concentrations of BADGE if the hemocytes are already primed for cell proliferation by the expression of dominant active Ras GTPase. We conclude that chronic exposure to the xenobiotic BADGE throughout development can induce cell proliferation.

**Electronic supplementary material:**

The online version of this article (10.1007/s11356-020-08899-7) contains supplementary material, which is available to authorized users.

## Introduction

By 2015, the global production of plastics reached 380 million metric tons annually (Geyer et al. 2017; Jambeck et al. 2015) and the tonnage continues to increase. Bisphenols, which have been in commercial use as plasticizers since the 1950s, are reported as estrogenic mimics and may interfere with hormonal activity, especially during gestational and early developmental stages. Furthermore, several bisphenols have been shown to have neurotoxic properties or are proposed to be carcinogenic (Jadhav et al. 2017; Pang et al. 2019). Bisphenol A diglycidyl ether (BADGE), an epoxy resin produced through the reaction of bisphenol A (BPA) and epichlorohydrin, is found in canned food and beverages, as well as in paints and adhesives, where it is used as an internal varnish to prevent degradation (Poole et al. 2004). During the manufacturing process there is some residual BADGE not covalently bound to the epoxy resin. This means the pollutant can easily leach from the container to contaminate food and beverages. Moreover, upon contact with aqueous or acidic matrices, BADGE can be hydrolyzed to form derivatives, such as BADGE·H_2_O and BADGE·2H_2_O (Suarez et al. 2000). Additionally, the presence of hydrochloric acid in organosol lacquers promotes the formation of chlorohydroxy derivatives, including BADGE·2HCl, BADGE·HCl, and BADGE·H_2_O·HCl (Hammarling et al. 2010). Many of these derivatives have been shown to induce both cytotoxic and genotoxic effects (Suarez et al. 2000).

Even though BADGE is manufactured from BPA, studies indicate that when BADGE is metabolized, it is not a significant source of BPA (Climie et al. 1981). BADGE and its hydrolytic derivatives have been found in human urine, plasma, and adipose fat (Wang et al. 2012; Wang et al. 2015). Due to the fact that BADGE is easily hydrolyzed in water or chlorinated in chlorine-containing coating solutions (Yonekubo et al. 2008), its concentration is difficult to detect in epoxy-coated commodities, and therefore, its disrupting effects can be underestimated. Similar to BPA, the estrogenic activity and reproductive/developmental toxicity of BADGE have been demonstrated (Hyoung et al. 2007; Nakazawa et al. 2002).

The toxicity of the BPA has been extensively studied, yet current information on the physiological and molecular effects of BADGE is scarce (Poole et al. 2004). Furthermore, the reported effects of BADGE on cell proliferation have been contradictory. For instance, BADGE was reported to decrease cell proliferation and induce morphological changes, as well as cell detachment, in human colorectal adenocarcinoma Caco-2 cells (Ramilo et al. 2006). On the other hand, BADGE, and its chlorohydroxy derivatives, triggered proliferation in human breast cancer T47D cells (Nakazawa et al. 2002). There is also evidence that through its inhibition of the nuclear receptor peroxisome proliferator–activated receptor-gamma (PPARγ) BADGE may drive cells towards proliferation at the exclusion of cellular differentiation (Zhu et al. 2013).

Most studies on the effects of BADGE have been performed in cell lines. In our study, we employed the model organism *Drosophila melanogaster* as the experimental subject to gain a better understanding of the xenobiotic effects of BADGE exposure in vivo. To do this, we performed whole transcriptome sequencing on RNA from flies exposed to BADGE throughout development. This provided a number of lead candidate genes involved in the effect of BADGE and several of these leads were verified in expression analysis. Moreover, we performed physiological studies providing further functional information on the effect of BADGE.

## Materials and methods

### Fly strain and maintenance

The *w*^*1118*^*; P {w[+mC] = Hml-GAL4.Delta}2, P {w[+mC] = UAS-2xEGFP}AH2 (Hml-GFP)* and w*; P {w[+mC] = UAS-Ras85D.V12}2, (UAS-Ras^*V12*^*)* strains were provided by the Bloomington Drosophila Stock Center (Bloomington, IN, USA). The *CantonS* and *OregonR-C* flies, also obtained from the Bloomington Drosophila Stock Center, were crossed to create the *CSORC* laboratory wild-type strain. All fly stocks were raised on Jazz-mix *Drosophila* food (Fisher Scientific) and supplemented with yeast extract (Genesee Scientific, San Diego, CA, USA). The cultures were maintained at 25 °C and 60% humidity on a 12:12 light/dark cycle. The adult male flies for experiments, unless otherwise stated, were collected after eclosion and aged for 5–7 days. Since the *Hml-GFP* flies express both green fluorescent protein and GAL4 in hemocytes, the *Hml-GFP* strain was crossed to *UAS-Ras*^*V12*^ to overexpress *Ras*. For examining the hemocyte by bleeding assay, no further staining was applied.

### Bisphenol A diglycidyl ether feeding

The BADGE (Sigma-Aldrich, Stockholm, Sweden) solutions were dissolved in 99% ethanol, and either 1 ml of BADGE solutions or ethanol was diluted in 50 ml Jazz-mix *Drosophila* food. The final BADGE concentrations in fly food were 20 μM (or 135 μM for live imaging), 60 μM, and 200 μM, respectively. After BADGE-enriched food preparation, the female and male flies (3:1 ratio) were placed in pre-prepared bottles and allowed to lay eggs on the food. The larvae were continuously raised on BADGE-enriched or control food until they eclosed. When the progeny eclosed, male flies were collected and transferred to a vial with 6 ml of BADGE-enriched food for further uses.

### mRNA preparation and SOLiD sequencing

The mRNA was isolated using Dynabeads mRNA purification kit (Thermo Fisher, Sweden) (Williams et al. 2016). The flies used for sequencing were fed on either control or 20 μM BADGE-enriched food. Three biological replicates, which contained 10 whole flies in each replicate, were prepared for sequencing. As we described previously (Williams et al. 2016), the RNA-seq data were analyzed using TopHat and Cufflinks. To test the differential expression of genes, the pre-analyzed data were further evaluated using the Cuffcompare and Cuffdiff tools. The calculated *p* value and *q* value (the FDR-adjusted *p* value of the test statistic) from Cuffdiff were used. Only genes with *q* < 0.05 and log2 > ±1-fold change were considered as significantly affected.

### RNA purification, cDNA synthesis, and qRT-PCR

#### RNA

The phenol-chloroform method was used for RNA extraction from tissue samples (Williams et al. 2013). Ten whole male flies, 5–7 days old, were homogenized with 800 μl TRIzol (Thermo Fisher, Sweden), 200 μl chloroform (Sigma-Aldrich, Sweden) was added, and samples were centrifuged at 12000 rpm for 15 min at 4 °C. The aqueous layer, which contained RNA, was separated and 500 μl isopropanol (Solvaco AB, Sweden) was added. The RNA was precipitated by storing the samples at − 32 °C for 2 h. Samples were centrifuged at 12,000 rpm for 10 min at 4 °C, to collect the RNA pellets, which were then washed with 75% ethanol (Solvaco AB, Sweden) to remove the organic impurities. Samples were allowed to air dry to remove any traces of ethanol. Dried RNA pellets were dissolved in 21.4 μl of RNAse-free water (Qiagen GmBH, Germany) and 2.6 μl of DNAse incubation buffer (Roche GmBH, Germany). The samples were incubated at 75 °C for 15 min to ensure complete dissolution of RNA-pellets. Two microliters of DNAse I (10 U/μL, Roche GmBH, Germany) was added to each sample and incubated at 37 °C for 3 h to remove DNA contamination. DNAse was deactivated by incubating the samples at 75 °C for 15 min. The removal of DNA was confirmed by PCR using Taq polymerase (5 U/μl, Biotools B & M Labs, Spain), followed by agarose gel electrophoresis. The RNA concentration was measured using a nanodrop ND 1000 spectrophotometer (Saveen Werner, Sweden).

### cDNA synthesis

cDNA was synthesized from RNA template using dNTP 20 mM (Fermentas Life Science, USA), random hexamer primers, and M-MLV reverse transcriptase (200 U/μl, Invitrogen, USA) by following the manufacturer’s instructions. cDNA synthesis was confirmed by PCR followed by agarose gel electrophoresis (Williams et al. 2013).

### qRT-PCR

Relative expression levels of three housekeeping genes (Rp49 and RpL11) and of the genes of interest were determined with quantitative RT-PCR (qPCR) (Williams et al. 2013). Each reaction, with a total volume of 20 μl, contained 20 mM Tris/HCl pH 9.0, 50 mM KCl, 4 mM MgCl_2_, 0.2 mM dNTP, DMSO (1:20), and SYBR Green (1:50000). The template concentration was 5 ng/μl and the concentration of each primer was 2 pmol/μl. Primers were designed with Beacon Designer (Premier Biosoft, CA, USA) using the SYBR Green settings. All qPCR experiments were performed in duplicate; for each primer pair, a negative control with water and a positive control with 5 ng/μl of genomic DNA were included on each plate. Amplifications were performed with 0.02 μg/ml Taq DNA polymerase (Biotools, Sweden) under the following conditions: initial denaturation at 95 °C for 3 min, 50 cycles of denaturing at 95 °C for 15 s, annealing at 52.8–60.1 °C for 15 s, and extension at 72 °C for 30 s. Analysis of qPCR data was performed using MyIQ 1.0 software (Bio-Rad) as previously reported. Primer efficiencies were calculated using LinRegPCR (Ramakers et al. 2003) and samples were corrected for differences in primer efficiencies. The GeNorm protocol described by Vandesompele et al. (Vandesompele et al. 2002) was used to calculate normalization factors from the expression levels of the housekeeping genes. Differences in gene expression between groups were analyzed with ANOVA followed by Fisher’s PLSD test where appropriate. *p* < 0.05 was used as the criterion of statistical significance. The following primers (Thermo Fisher Scientific, Germany) *Rp49*-F: 5′- CACACCAAATCTTACAAAATGTGTGA-3′ *Rp49*-R: 5′-AATCCGGCCTTGCACATG-3’ *CycB*-F: 5′-GTGAACGAGCCCACCTTAAAG-3′ *CycB*-R: 5′-GAAACTCCCATCACGGGTTTG-3′ *CycE*-F: 5′-AGCCTCCATCAGCTAAGCG-3′ *CycE*-R: 5′-GCAACCGATGACAGATTGCC-3′ *stg*-F: 5′-CAGCAGTTCGAGTAGCATCAA-3′ *stg*-R: 5′-CTCCCATAGCTGGCAGAATTTT-3′ *png*-F: 5′-GCCAGGGCAAGGTGGATTT-3′ *png*-R: 5′-CCACACGGGATCGTAGAGAC-3′ *gnu*-F: 5′-TAAATGGGGAGACCAGGCTAC-3′ *gnu*-R: 5′-CGCACTTTTCAACTTGGATTCCT-3′ *plu*-F: 5′-GGAATACCGCCCTATTGAAGG-3′*plu*-R: 5′-ATCTTCGCCTCAACAGTTCCT-3′.

#### Larvae bleeding and hemocytes counting

Third instar larvae were collected and kept in 1x PBS solution for 5–10 min before bleeding. One drop of 1x PBS solution was placed on a glass slide. To get enough hemocytes, the larva was squeezed by another forceps. After 30 min, the cells were settled and attached to slide. For the *CSORC* larvae, the hemocytes were stained by DAPI for 20 min. For the *Hml-GFP* > *UAS-Ras*^*V12*^ cross, the flies were raised at 20 °C in order to inhibit the activity and lethal effect of *Ras* overexpression. The slides were then mounted and the images were taken by a Zeiss Axioplan 2 microscope. The number of hemocytes was counted using the ImageJ software.

#### Live imaging

*Hml-GFP* larvae were used for live imaging and maintained on 0, 60, 135, and 200 μM BADGE food, respectively. Third instar larvae were anesthetized on ice and fixed by a drop transparent nail polish. The slides were immediately imaged using a Zeiss LSM 700 confocal microscope.

#### Data analysis

The data analysis and plotting were performed by Prism Graphpad 5. The data were plotted as mean ± SEM. Normal distribution was performed using the Kolmogorov-Smirnov test of normality. All the significant differences were tested following one-way ANOVA with Tukey’s post hoc. The significances were represented as *p < 0.05, **p < 0.01, and ***p < 0.001 and also specified in the corresponding figure legend.

## Results

### BADGE exposure causes upregulation of cell cycle genes

To gain a better understanding of how bisphenol A diglycidyl ether (BADGE) influences biological systems in vivo, F_0_*Drosophila* females were allowed to lay eggs on food containing 20 μM BADGE (calculated from the human no observed adverse effect level, NOAEL) (Poole et al. 2004). Three replicates of F_1_ progeny were collected, which had been raised on BADGE-containing food from embryos to 5–7-day-old adults. Total RNA was then extracted from male flies for whole transcriptome sequencing. Due to the fact that the reproductive cycle of female flies could potentially influence their behavior, including food intake, we used only adult males in our experiments. By mapping the *Drosophila* transcriptome to the reference genome obtained from FlyBase (build dmel_r5.47_FB2012_05), 15,147 transcripts were identified, including expressed genes (mRNA), miRNA, snRNA, snoRNA, and tRNA. After performing pair-wise comparisons between all sequenced replicates and taking into consideration the “false discovery rate” and correcting for it using the Benjamini-Hochberg correction, 338 genes were differentially expressed (Supplementary Dataset S1).

**Fig. 1:**
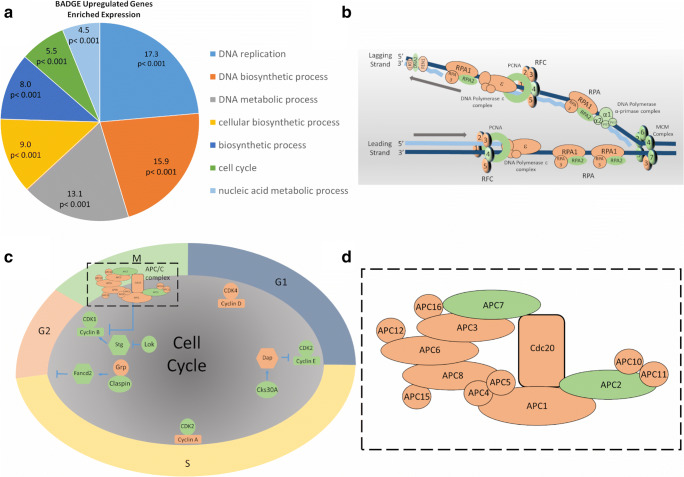
BADGE increases transcript numbers of genes linked to cell proliferation. **a** Pie chart showing PANTHER classification of genes whose transcript number significantly increased in male *Drosophila* upon chronic BADGE exposure throughout development (number indicates the percentage of total BADGE up- or downregulated genes, *p* value indicates this is significant when compared with the total number of genes in the genome linked to this category). **b** Many of the genes with increased transcript numbers were linked to DNA replication. **c** Another group of genes whose transcript number was significantly increased in flies raised on BADGE-containing food was those regulating cell cycle. **d** Blow-up of the APC complex outlined with a dashed box in (**c**). **b**–**d** Proteins in green bubbles were transcriptionally enhanced after BADGE feeding while those in orange were not significantly affected

Using PANTHER and DAVID, we categorized the differentially expressed genes by function (Huang da et al. 2009a, b; Mi et al. 2013). Interestingly, flies raised on food containing BADGE at the NOAEL level showed a notable enrichment in genes connected with DNA proliferation and cell cycle regulation (Fig. 1a–d and Table 1). Among the DNA proliferation genes having a significant increase in transcript copy number, Pol α-primase and mini-chromosome maintenance (Mcm) genes were highly enriched (Fig. 1b and Table 1). Many cell cycle genes having a significant increase in transcript copy number were linked to the control of G_1_-S and G_2_-M transitions (Fig. 1c, d and Table 1).

**Table 1:** Genes whose transcript copy number was significantly increased in *Drosophila* males raised on BADGE-containing food throughout the development

Gene ID	Symbol	Human ortholog	Log2 (fold_change)
DNA proliferation			
FBgn0020633	Mcm7	MCM7	3.24
FBgn0017577	Mcm5	MCM5	3.06
FBgn0014861	Mcm2	MCM2	2.74
FBgn0040290	RecQ4	RECQL4	2.52
FBgn0005655	PCNA	PCNA	2.32
FBgn0031540	Pif1	PIF1	2.26
FBgn0030170	CG2990	DNA2	2.05
FBgn0259113	DNApol-alpha180	POLA1	2.02
FBgn0025815	Mcm6	MCM6	1.85
FBgn0005696	DNApol-alpha73	POLA2	1.83
FBgn0259676	DNApol-alpha60	PRIM2	1.81
FBgn0086695	hd	DONSON	1.63
FBgn0043002	Chrac-14	POLE3	1.43
FBgn0015929	dpa	MCM4	1.41
FBgn0260985	RfC4	RFC4	1.4
FBgn0032906	RPA2	RPA2	1.31
FBgn0027903	Pol31	POLD2	1.15
Cell cycle regulation			
FBgn0001120	gnu		7.23
FBgn0010097	gammaTub37C	TUBB3	5.73
FBgn0000927	fs(1)Ya		4.77
FBgn0003525	stg	CDC25C	4.19
FBgn0052251	Claspin	CLSPN	3.94
FBgn0000826	png	NEK8	3.76
FBgn0029879	APC7	ANAPC7	2.83
FBgn0019686	lok	CHEK2	2.41
FBgn0004107	Cdk2	CDK2	2.13
FBgn0013548	l(2)dtl	DTL	2.03
FBgn0000405	CycB	CCNB1	1.81
FBgn0003114	plu	POTEA	1.69
FBgn0034403	CG18190	MAPRE2	1.56
FBgn0010382	CycE	CCNE1	1.52
FBgn0051658	Nnf1b		1.35
FBgn0032105	borr	CDCA8	1.24
FBgn0038827	Fancd2	FANCD2	1.20
FBgn0004106	Cdk1	CDK1	1.17
FBgn0039638	dgt6	HAUS6	1.17
FBgn0264296	CG43774	ANAPC2	1.14
FBgn0263979	Caf1–55	RBBP4	1.05
FBgn0030500	Ndc80	NDC80	1.04
FBgn0010314	Cks30A	CKS2	1.02

By using PANTHER and DAVID to analyze genes having a significant decrease in transcript copy number, we were able to determine the largest set belonged to the cellular response to light stimulus and the response to oxygen levels (Supplementary Figure 1). Furthermore, many genes with a significant decrease in copy number were involved in regulating metabolism or neuronal interactions (Supplementary Figure 1).

### BADGE feeding interferes with cell cycle genes at the transcriptional level

**Fig. 2:**
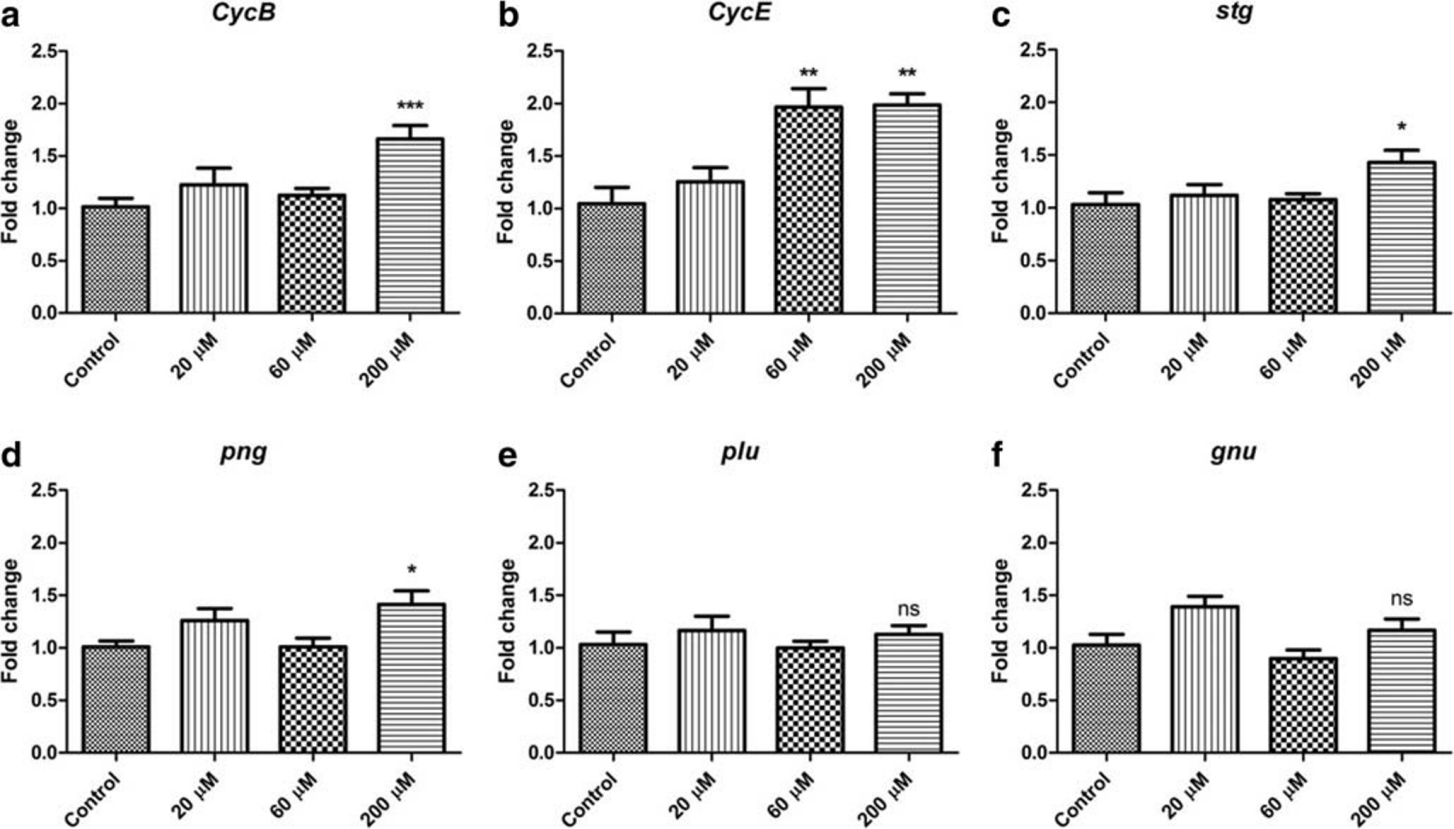
BADGE feeding causes an increase in cell cycle gene expression. *CSORC* flies were raised on either ethanol-control food or BADGE food and aged for 5–7 days before sampling. The flies were kept at – 80 °C for long-term storage if necessary. RNA was carefully extracted from whole flies and converted to cDNA before performing qRT-PCR (*n* = 6 biological replicates for each treatment and 10 male flies were used for each biological sample). Data showing that *CycB* (**a**), *stg* (**c**), and *png* (**d**) were notably increased in 200 μM BADGE-fed flies and *CycE* (**b**) was increased in both 60 μM and 200 μM BADGE-fed flies. However, the expressions of *plu* (**e**) and *gnu* (**f**) were not significantly influenced by BADGE (*p < 0.05, ***p < 0.005 compared with ethanol-control feeding, one-way ANOVA with Tukey’s post hoc test for multiple comparisons was applied. Error bar represented SEM)

To confirm the effect of BADGE on cell cycle, we selectively tested the transcriptional expression of some important cell cycle regulatory genes that had a significant increase in transcript copy number in our sequencing experiment. Flies were maintained throughout the development on three BADGE dosages, 20 μM, 60 μM, and 200 μM, as well as control food. As before, F_1_ males were collected and aged 5–7 days before sampling. Consistent with the transcriptome sequencing data, transcripts of *Cyclin B* (*CycB*), *pan gu* (*png*), and *string* (*stg*) were significantly increased in flies maintained on food containing 200 μM BADGE (approximately 50%) (Fig. 2a, c, d). *Cyclin E* (*CycE*) transcript was significantly increased in flies raised on both 60 μM and 200 μM BADGE (Fig. 2b). However, the transcript levels of two components of the *png*-kinase complex, *gluon* (*glu*) and *giant nuclei* (*gnu*), were not changed (Fig. 2e, f). The inconsistency can result from the different sensitivities and procedures between whole transcriptome sequencing and quantitative RT-PCR (qPCR). Together, the two methodologies, sequencing and qPCR, verified that chronic exposure to BADGE during development upregulates cell cycle genes in *Drosophila*.

### BADGE exposure leads to over-proliferation of larval hemocytes

**Fig. 3:**
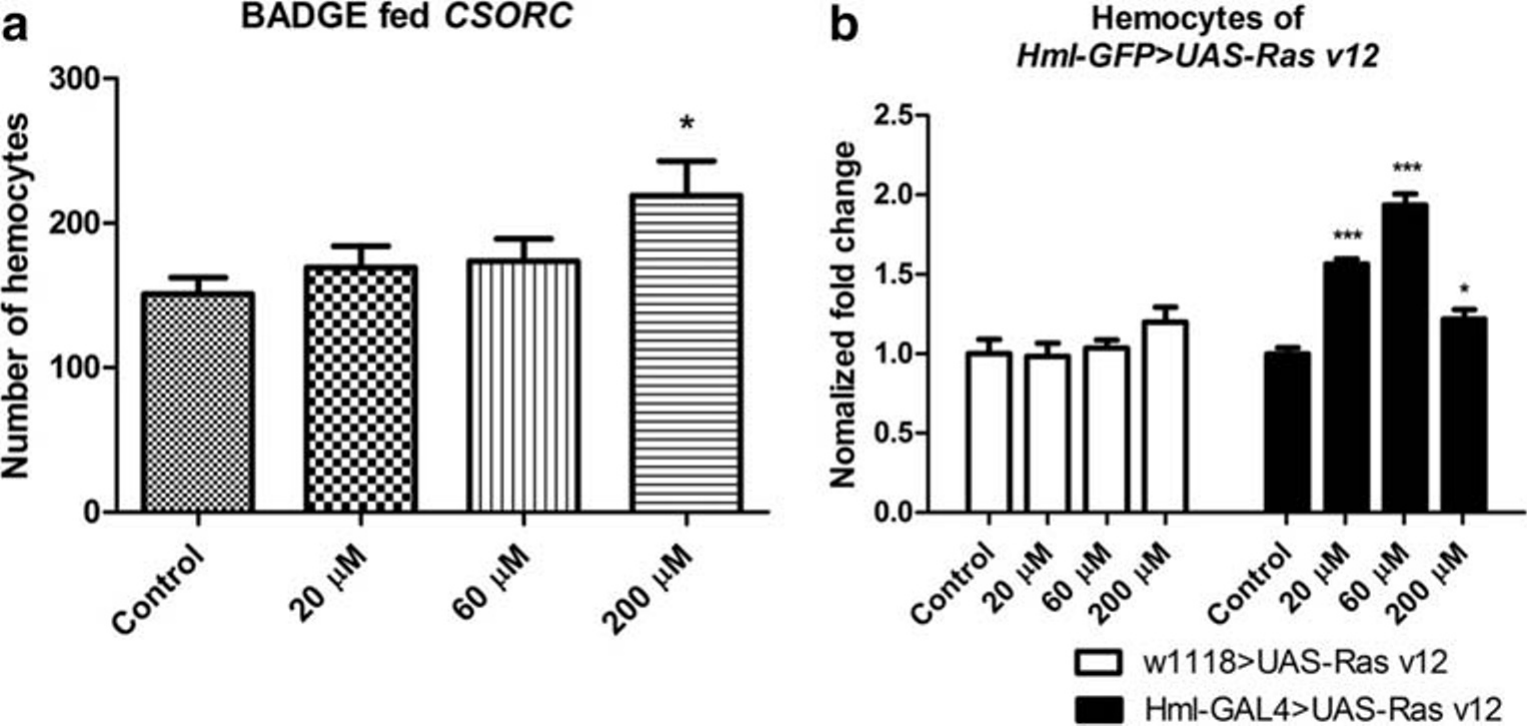
BADGE causes over-proliferation of larval hemocytes. We employed the hematopoietic system to examine the effect of BADGE inducing cell over-proliferation in vivo. Third instar larvae were selected to perform the bleeding assay. **a***CSORC* larvae were bled to test if BADGE can cause significant cell proliferation. At least 15 larvae were bled and the number of hemocytes was counted for each concentration. Compared with the controls, larvae fed on 200 μM BADGE had significantly more hemocytes (~ 40%) while 20 μM and 60 μM were not influenced. **b** To confirm our result, we employed the GAL4-UAS system by crossing *Hml-GFP* with *UAS-Ras*^*V12*^ to overexpress *Ras* in blood cells. Additionally, all data were normalized to corresponding control fed flies, which could eliminate the difference between hemocyte amounts between the two groups. After BADGE diet and bleeding, the *Hml-GFP > UAS-Ras*^*V12*^ flies, compared with *w*^*1118*^ *> UAS-Ras*^*V12*^ controls, showed an obvious increase in the number of circulating hemocytes on 20 μM, 60 μM, and 200 μM BADGE food (*p < 0.05, ***p < 0.005 compared with ethanol-controls, one-way ANOVA with Tukey’s post hoc test for multiple comparisons was applied. Error bars represent SEM)

The *Drosophila* larval hematopoietic system is suitable for assessing cell proliferation (Zettervall et al. 2004). Therefore, we employed this system to test the effect of BADGE on cell proliferation. As with the whole transcriptome sequencing, we used our laboratory wild-type *CSORC* strain and maintained the larvae on BADGE-containing food. All food, including for controls, was dyed with red food coloring. In this way, we were able to distinguish early and late wandering third instar larvae, which is important when comparing circulating hemocyte numbers (Zettervall et al. 2004). The number of circulating hemocytes increases rapidly during development; therefore, by using the red food coloring, we were able to stage wandering larvae according to the presence (early wandering third instar larvae) or absence (late wandering third instar larvae) of food in the gut (Zettervall et al. 2004). Late wandering third instar larvae were bled on a glass slide and stained with DAPI to visualize the nuclei. ImageJ was then employed to count the number of hemocytes (Howell et al. 2012). Compared with control flies, larvae raised on 20 μM and 60 μM BADGE did not induce a significant increase in the number of circulating hemocytes. However, raising larvae on food containing 200 μM BADGE caused a 42% increase (*p* < 0.05) in the number of circulating hemocytes (Fig. 3a).

Next, we employed the GAL4-UAS system to sensitize hemocyte proliferation in larvae. The *Hml-GFP* driver, which expresses both GAL4 and GFP specifically in hemocytes (Goto et al. 2003), was crossed to the *UAS-Ras*^*V12*^ strain, to overexpress constitutively active Ras GTPase. This should cause the *Hml-GFP > UAS-Ras*^*V12*^ larvae to be sensitive to agents that influence proliferation (Asha et al. 2003). In line with our assumption, unlike wild-type larvae, where hemocyte numbers only significantly increased when flies were raised on food containing 200 μM BADGE, the *Hml-GFP > UAS-Ras*^*v12*^ larvae exhibited significant cell over-proliferation at lower concentrations (20 μM and 60 μM), when compared with *Hml-GFP > UAS-Ras*^*v12*^ larvae raised on normal food (Fig. 3b). In fact, the normalized hemocyte fold-changes in circulating cell numbers were elevated approximately 60% (*p* < 0.005) and 100% (*p* < 0.005), respectively (Fig. 3b). Again, this increase is compared with *Hml-GFP > UAS-Ras*^*v12*^ larvae raised on normal food, which have significantly higher numbers of circulating hemocytes than wild type larvae (Asha et al. 2003).

### BADGE does not damage hematopoietic tissue

**Fig. 4:**
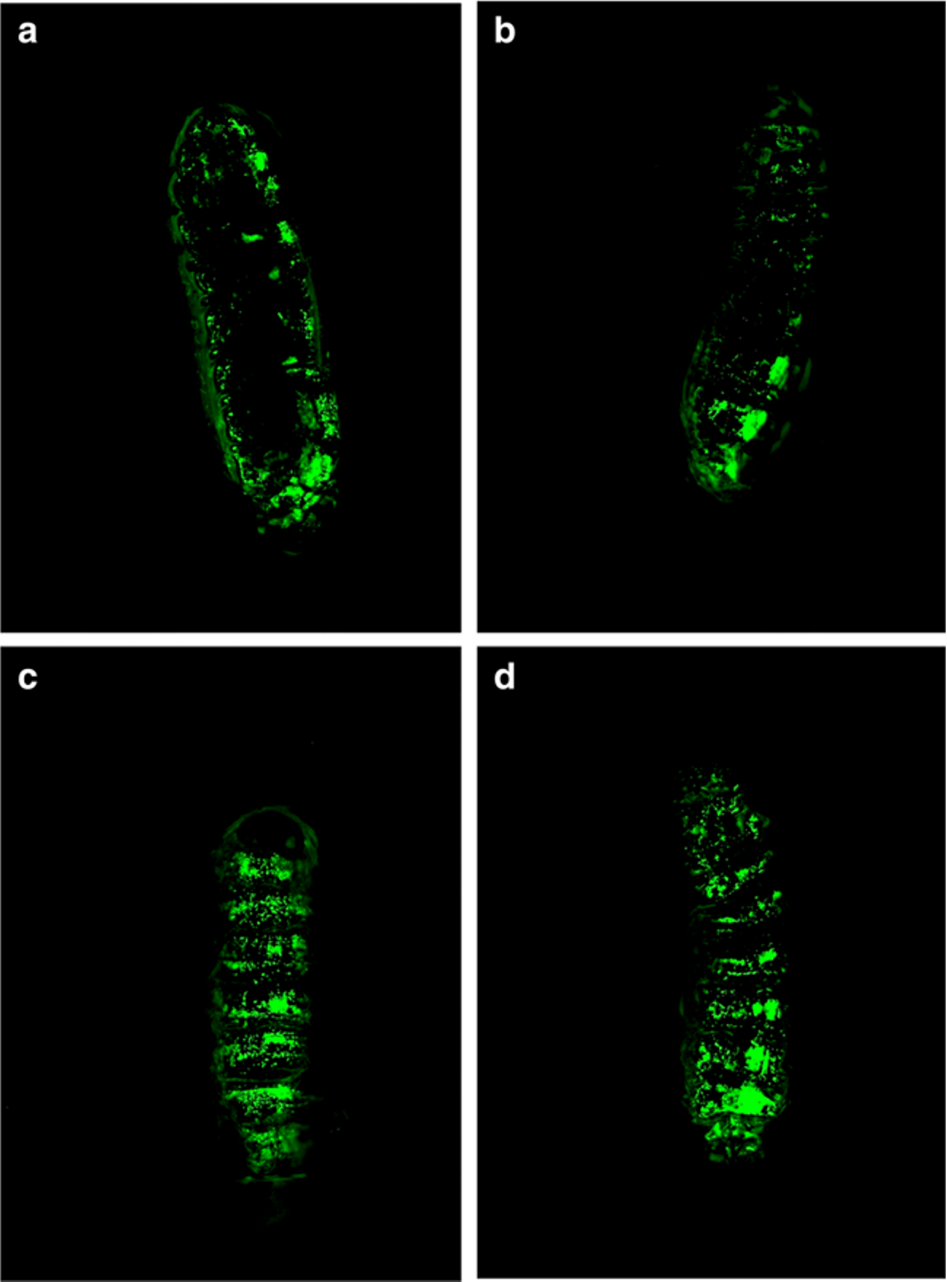
BADGE does not disturb the sessile hemocyte population. The increase in circulating hemocytes can be caused by the release of a sessile population attached to the epidermis; therefore, we used confocal microscopy to evaluate the effect of BADGE on this hematopoietic tissue. The *Hml-GFP* flies were raised and allowed to lay eggs on either BADGE or control food. Living F_1_ larvae were anesthetized on ice and fix by nail polish on a glass slide. Images were taken immediately. Data show larvae raise on food containing 0 μM (**a**), 60 μM (**b**), 135 μM (**c**), and 200 μM (**d**) BADGE. No morphological changes were observed in the sessile population; the sessile bands were clear and intact in each larval segment

We performed live imaging with *Hml-GFP* larvae to exclude the possibility that BADGE induces the release of a pool of sessile hemocytes attached to the epidermis in each larval segment. Since there is a GFP signal in the hemocytes, these cells are easily visible under fluorescent microscopy. For this experiment, we exchanged the NOAEL mimicking 20 μM concentration for larvae raised on food containing 135 μM BADGE, in order to understand if BADGE was capable of disrupting the hematopoietic tissue. Consequently, *Hml-GFP* larvae were raised on food containing 0 μM (Fig. 4a), 60 μM (Fig. 4b), 135 μM (Fig. 4c), or 200 μM (Fig. 4d) of BADGE. At all BADGE concentrations, the morphological structure of the segmentally defined epidermis-attached sessile hemocytes was intact (Fig. 4b–d).

## Discussion

In Drosophila males, chronic exposure to BADGE throughout developmental causes a significant change in the transcript number of 338 genes. The expressions of genes connected to DNA polymerization and cell cycle control were particularly enriched among these transcripts. Many genes whose transcript number significantly decreased were connected to neuronal signaling and synapsis homeostasis. For a subset of genes connected to cell cycle control, we confirmed the increased transcript number by using quantitative real-time PCR. In relation to the significant number of cell proliferation genes with increased transcript number, we also demonstrate that late third instar larvae raised on BADGE-containing food have a significantly increased number of circulating hemocytes, without any obvious physical lesion of a hematopoietic tissue, known as the lymph gland in *Drosophila*, or a pool of sessile hemocytes attached to the larval epidermis. Furthermore, the susceptibility of the hematopoietic system to BADGE exposure can be sensitized in a background where dominant active Ras GTPase is expressed specifically in hemocytes.

In the current study, we applied both genetic and phenotypic studies to reveal the cell proliferative effect of chronic BADGE exposure at a level comparative to the human NOAEL. Of note, in a breast cancer cell line (T47D), it was observed that BADGE induced cellular proliferation (Nakazawa et al. 2002). Other studies have also hinted at the proliferative effects of BADGE exposure in cell lines (Suarez et al. 2000; Sueiro et al. 2006). However, in all of those studies, in vivo data was missing. Interestingly, our whole transcriptome sequencing data, using adult male *Drosophila*, demonstrates that exposure to BADGE throughout development causes a significant increase in the transcript number of genes linked to DNA replication and cell cycle control (see Fig. 1). For example, the expressions of *CycB*, *CycE*, and *stg* genes are significantly increased, which gives hints that cell replication may be abnormal.

We provide in vivo phenotypic evidence demonstrating the ability of BADGE to induce cell proliferation. In recent years, the *Drosophila* hematopoietic system has been increasingly used in inflammatory and cell proliferation–related studies (Milton et al. 2014; Wang et al. 2014; Zettervall et al. 2004). With the advantages of the hematopoietic system, we report a notable increase in the number of circulating hemocytes in late third instar larvae maintained on BADGE-containing food throughout development, which is consistent with the RNA-seq and qPCR results. This not only demonstrates that genes involved in regulating cell proliferation are transcriptionally more active, but also that cell proliferation in at least one system (hematopoietic) is significantly increased upon chronic exposure to BADGE. The peroxisome proliferator–activated receptor-gamma (PPARγ) has been linked to BADGE responses (Chen et al. 2019; Dworzanski et al. 2010; Nakamuta et al. 2002). However, there is no obvious PPARγ homolog in *Drosophila*, which suggests the involvement of additional molecular pathways that need to be identified in the future.

Although BADGE is detected at a low level in some epoxy resin–based products, the influence of chronic and developmental exposure needs to be considered. Some surveys reported that daily BADGE intake in humans is much lower than the NOAEL level of 15 mg/kg body weight/day (reviewed in (Poole et al. 2004)). However, we believe these results do not go against our study, as humans are exposed to multiple xenobiotic chemicals via various routes over an extended period of time. The continual interaction between different substrates may have an influence on the final exposure outcomes.

## Conclusions

We report that chronic BADGE exposure throughout development at a level equivalent to the human NOAEL increases the transcript copy number of genes related to cell proliferation and causes hemocyte proliferation in *Drosophila* larvae. Furthermore, we show that feeding BADGE to larvae whose cells are already sensitized for cell division by the expression of a dominant active Ras (Asha et al. 2003) significantly increased the number of circulating hemocytes at a BADGE concentration 100-fold lower than non-sensitized cells. Due to the ubiquitous exposure to such xenobiotics in modern society, it is important to elucidate a molecular mechanism for the effect of BADGE on cell proliferation in vivo.

## Electronic supplementary material

ESM 1 (DOCX 683 kb)
