# Supplementary material for: Exposure to the environmental pollutant bisphenol A diglycidyl ether (BADGE) causes cell over-proliferation in Drosophila
Source: Environ Sci Pollut Res Int. 2020 Apr 28;27(20):25261–70. doi: 10.1007/s11356-020-08899-7 (PMC7329772; doi:10.1007/s11356-020-08899-7)
Supplement: Supplementary file 1 — (DOCX 683 kb) [file 11356_2020_8899_MOESM1_ESM.docx]

**Supplementary Figure 1**

**
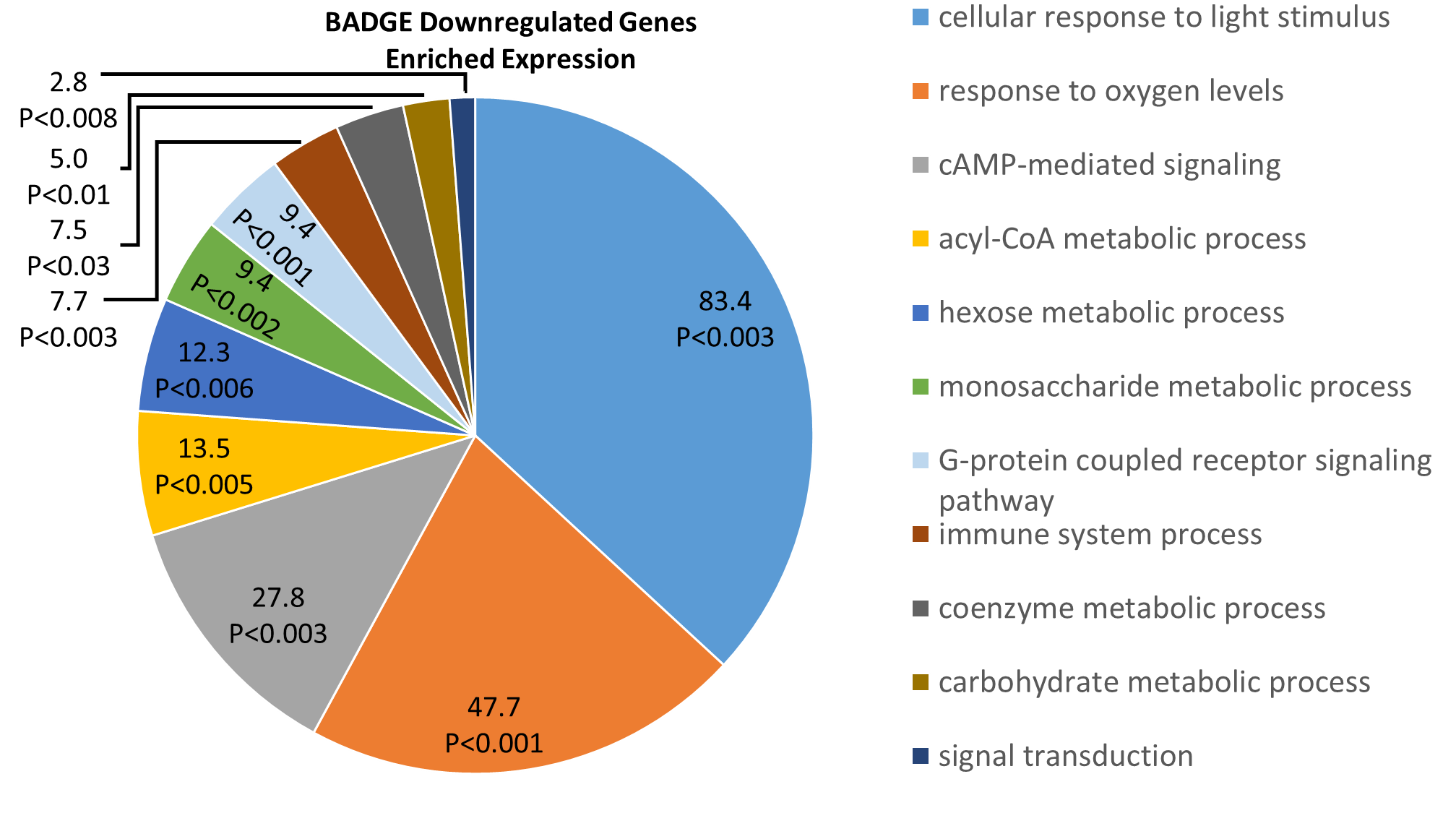
**

**Supplementary Figure 1: BADGE decreases transcript numbers of genes linked to neuronal signaling and metabolic pathways.** (A) Pie chart showing PANTHER classification of genes whose transcript number signficantly decreased in male *Drosophila* upon chronic BADGE exposure throughout development (Number indicates percentage of total BADGE up or down regulated genes, p value indicates this is signficant when compared to total number of genes in the gemone linked to this category).
